# Integrative Nursing Interventions for Cancer-Related Symptoms in Oncology Inpatients: Results of a Descriptive Pilot Study

**DOI:** 10.1177/15347354241239930

**Published:** 2024-04-29

**Authors:** Lea Raiber, Christian Raff, Johanna Thiele, Klaus Kramer

**Affiliations:** 1 University Hospital Ulm, Ulm, Germany; 2 Interprofessional Graduate School for Integrative Medicine and Health, Witten/Herdecke University, Germany

**Keywords:** supportive care, integrative oncology, integrative nursing, nonpharmacological intervention, cancer patients, integrative medicine, nursing intervention, symptom management, descriptive study, complementary medicine

## Abstract

**Background:**

Integrative nursing (IN) is an essential component of integrative medicine and integrative oncology. IN includes a range of external naturopathic, integrative nursing interventions, such as compresses, embrocation, and foot/hand baths, aimed at alleviating symptoms and side effects of conventional treatment. The project IMPLEMENT-UKU (“Implementation of IN at the University Hospital Ulm”) was accompanied by a descriptive pilot study on the use of IN interventions on cancer-related symptoms in oncology inpatients, the characterization of these patients and the evaluation of the impact.

**Methods:**

A single-arm study was conducted using a paper-based questionnaire administered before the IN interventions (t0) and 24 hours after the IN interventions (t1). Topics included sociodemographic data, symptoms, quality of life, health status, psychological burden, attitudes, and experience and satisfaction with the IN interventions. Analyses were descriptive using absolute and relative frequencies.

**Results:**

During the 6-month study period out of 66 patients recommended for IN consultation by medical and nursing staff on 2 wards, 62 (93.9%) accepted the offer. Of those patients who received IN, 21 patients (33.9%) participated in the study. The number of IN interventions received per patient ranged from 1 to 3 during the 24-hour survey period. And a total of 36 IN interventions were performed: The most treated body region was the feet/legs (50.0%), followed by the back (25.0%), using oils such as solum oil (41.7%) and lavender oil (13.9%). Embrocation (77.8%) was the predominant type of IN intervention. For patients, the mean FACIT-F score was 29.2 ± 12.5. The mean PHQ-4 score was 3.0 ± 1.9. Regarding sleep quality in the last 4 weeks, 13 participants (61.9%) described it as rather or very poor. Satisfaction with the IN was high, with a large proportion of participants evaluating the IN interventions very positively.

**Conclusion:**

The study’s findings suggest that there is a great need for IN among oncology inpatients. These patients are open to and interested in IN interventions and evaluate them positively. IN provides a promising opportunity to provide non-pharmacological support to inpatients. The integration of IN in conventional oncology care settings may enhance patient-centered care and contribute to improved patient wellbeing.

## Introduction

Interest in integrative medicine is experiencing global momentum, with an increasing adoption among diverse patient populations.^1,2^ For instance, a comprehensive systematic review reveals that, on average, 51% of individuals diagnosed with cancer incorporate complementary and alternative medicine (CAM) into their treatment journey, encompassing a wide-ranging prevalence from 16.5% to 93.4%.^2^ Similarly, an extensive study conducted at German university hospitals indicates that 48% of hospitalized patients acknowledge either current or previous utilization of CAM as a supplementary approach to managing their present condition.^3^ Within the field of CAM, non-pharmacological interventions assume a pivotal role and are often administered or guided by skilled nursing professionals.^4^

In the context of integrative medicine and integrative oncology, integrative nursing (IN) is an essential component.^5-8^ IN interventions encompass a range of external naturopathic nursing interventions, such as compresses, embrocation, and foot/hand baths^9^ with essential oils, aimed at preventing and managing symptoms and side effects of conventional treatment.^8,10^ These interventions are particularly indicated for addressing common issues experienced by (oncology) patients, including sleep disorders, mucositis, and nausea.^11^ IN considers the patient′s individuality and personal experience of illness, with a focus on meeting their specific needs.^12^ A systematic review by Mühlenpfordt et al highlights the potential benefits of these external applications as supportive care, showing their suitability for improving overall condition, alleviating symptoms, and promoting psychological activation and relaxation.^13^

While there is internal evidence for IN interventions based on experiential knowledge and traditional expertise,^14^ external scientific evidence is currently limited. Some evidence exists regarding the effectiveness of specific, complementary interventions, such as aromatherapy for anxiety management,^15^ massage for coping with negative emotions and fatigue,^16^ or acupuncture for chemotherapy-induced nausea and vomiting.^17^ However, a gap still exists between experiential knowledge and study evidence, and various research groups are actively working to generate more evidence for IN interventions.^18^ Clinical guidelines have already been developed based on reviews and consensus processes, such as those for mucositis,^19^ pain^20^ and chemotherapy-induced peripheral neuropathy.^21^

The current project IMPLEMENT-UKU (“Implemen-tation of IN at the University Hospital Ulm”) offers IN as a consultation service for inpatients. Patients were provided with IN interventions throughout the entire hospital stay, in addition to their routine care, based on their symptom burden and preferences. The implementation of the project will undergo scientific evaluation, considering the perceptions and experiences of patients, their relatives, and the hospital staff. This presented article primarily focuses on the use of IN interventions for cancer-related symptoms in oncology inpatients, the characterization of these patients, and the evaluation of the impact.

The objectives of this study are the following:

(a) Investigate the acceptance, openness, and interest in the use of IN for cancer-related symptoms throughout the hospital stay,
(b) Characterize the inpatients, who receive IN and assess their burden,
(c) Evaluate perceptions and experiences related to the use of IN.

## Methods

### IMPLEMENT-UKU Project

As part of the project, patients received IN interventions as supportive care in form of counseling during their hospital stay. Patients were referred to the IN consultation by the nursing and medical staff on 2 oncology wards. Patients had to present with relevant symptoms indicating the need for IN with non-pharmacological interventions. Staff were trained in the referral process during ward meetings. Referred patients were then scheduled for an initial consultation and their first and subsequent IN sessions. If necessary, the patient is followed throughout during the entire hospital stay, resulting in a variable number of IN interventions per patient. Depending on the patient′s symptom burden and preferences, patients were offered different IN interventions in addition to their routine care. The IN interventions were performed by a nurse with 3-year vocational training and many years of professional experience, trained in complementary and integrative medicine with an additional IN qualification. The basis for offering the IN interventions was the IN catalog, which targets relevant cancer-related symptoms and is presented in Table 1. The IN catalog was developed using a combination of experiential knowledge, relevant literature,^9,15,20-22^ and existing recommendations from previous projects.^11,23^ The catalog underwent continuous revision and further development by the project working group to ensure its effectiveness and relevance.

**Table 1.** IN Catalog of IN Interventions in Use During the Study.

Symptom	Integrative Nursing Intervention^a^
Anxiety, sleep disorders, tension	• Embrocation with lavender oil^b^, solum oil^c^, mallow oil^c^, rose oil^b^ • Heart-compress with aurum-lavender oil cream^c^ • Liver-compress with hot yarrow infus • Hand-/foot-bath with lavender oil^b^
Respiratory complaints	• Diaphragm-compress with thyme oil^b^, solum oil^c^ • Embrocation with thyme oil^b^, solum oil^c^
Edema	• Compress with borago essence^d^ • Embrocation with rosemary oil^b^, solum oil^c^ • Therapeutic wash with citrus^d^
Pain	• Embrocation with aconite oil^c^, solum oil^c^
Exhaustion, weakness state, other side effects	• Liver-compress with hot yarrow infus • Hand-/Foot-bath with citrus • Embrocation with rose oil^b^, mallow oil^c^, solum oil^c^ • Therapeutic wash with citrus^d^, rosemary^d^
Digestive complaints	• Embrocation with chamomile oil^b^
Restlessness, rapid heartbeat	• Heart-compress with aurum-lavender oil cream^c^ • Wrist-compress with citrus oil^b^ • Embrocation with mallow oil, solum oil^c^, lavender oil^b^, rose oil^b^ • Hand-/foot-bath with lavender^d^

a Selected according to the patient’s symptom burden and preferences.

b Essential oils with a high-quality oil as a carrier: Lavender, Rose, Thyme, Rosemary, Chamomile, Citrus.

c Anthroposophic medicinal products: solum oil (*Aesculus hippocastanum e semine LA 25% sicc., Equisetum arvense ex herba LA 20%, Lavandulae aetheroleum, Solum uliginosum;* WALA, Germany); mallow oil (*Geranii aetheroleum, Malva arborea e floribus W 5%, Hypericum perforatum, Herba rec., Prunus spinosa e floribus W 5%, Sambucus nigra ex umbella W 5%, Tilia platyphyllos/cordata e floribus W 5%;* WALA, Germany); aconite oil (*Aconitum napellus e tubere ferm 33c Dil. D9 oleos., D-Campher, Lavandulae aetheroleum, Quarz Dil. D9 oleos.;* WALA, Germany); aurum-lavender oil cream (*Aurum metallicum praeparatum Dil. D4, Lavandulae aetheroleum, Aetheroleum extractum e floribus recentibus Rosae damascenae et centifoliae;* WALA, Germany).

d Other oil products: Borago essence (*Borago officinales ex herba LA 20%;* WALA, Germany), bath milk citrus, rosemary, lavender (Weleda, Germany).

### Study Design and Setting

A single-arm, descriptive pilot study was conducted using a paper-based questionnaire to assess the impact of IN. The monocentric study was conducted over a period of 6 months in 2022 at the University Hospital Ulm on 2 oncology wards. The study is an accompanying research: If the patient decides not to participate in the study, he or she still has the option to take up the IN offer within the IMPLEMENT-UKU project. The study received ethical approval from the Ethics Committee of the University of Ulm (488/21), Germany, and all participants provided written informed consent before enrolling in the study.

### Study Participants and Procedures

The study population represents a convenience sample of participants from the IMPLEMENT-UKU project. The purpose of the initial consultation was to inform patients about IN and the ongoing study. To be considered for inclusion in the study, patients had to be aged 18 years or older and have received the offer of IN. Non-inclusion criteria were cognitive deficits, immediate dying process, inability to provide informed consent, and insufficient knowledge of the German language.

The survey was administered before the IN interventions (t0) and 24 hours after the IN interventions (t1). One focus was on the baseline questionnaire (t0) to characterize patients receiving IN. Information on cancer-related symptoms was to be collected. The second questionnaire at 24 hours (t1) was designed to provide initial indications of the impact of reported symptoms as a short-term effect, and perception and satisfaction with IN. Participants were asked to independently complete the questionnaires. However, if self-completion was not possible, study staff, other than the attending nurse, were available to assist. In the context of a descriptive pilot study, the final sample size was determined by the practical feasibility of the number of consultations requested on the 2 wards during the 6-month study period.

### Assessment of Study Outcomes

The questionnaires comprised validated instruments supplemented with self-developed items. The topics covered sociodemographic data, symptoms, quality of life, health status, psychological burden, social support, exercise, attitudes, and experience and satisfaction with the IN. Fatigue levels were measured using the Functional Assessment of Chronic Illness Therapy – Fatigue (FACIT-F) with 13 items,^24^ which is also used for oncology patients.^25^ The scale ranges from 0 to 52, with higher scores indicating less fatigue. Sleep quality was assessed using items from 2 of the 7 components from the Pittsburgh Sleep Quality Index (PSQI),^26^ which is also used for oncology patients.^27^ The component “subjective sleep quality” was included with the question about the quality of sleep in the last 4 weeks, rated on a 4-point Likert scale from “very bad” to “very good.” In addition, all patients with poor sleep quality were asked in an item with a list of 10 possible reasons for the “sleep disturbance” component.

Psychological burden in terms of anxiety and depression was captured using the 4-item Patient Health Questionnaire for Depression and Anxiety (PHQ-4), which includes 2 subscales assessing depression and anxiety symptoms,^28^ and is used in oncology patients.^29^ The PHQ-4 score is categorized as normal (0-2), mild (3-5), moderate (6-8), and severe (9-12). The subscales range from 0 to 6, with scores ≥3 indicating clinically significant depression or anxiety.^30^ General life satisfaction was measured using the 1-item Short Scale Life Satisfaction (L-1).^31^ Mobility was assessed by patients using a numeric rating scale from 0, indicating extremely limited mobility, to 10, indicating no limitation. Subjective health was captured using a 5-point Likert scale ranging from “very good” to “very bad.” Additionally, patients rated their existing symptoms on a 5-point Likert scale from 1 (indicating “not at all”) to 5 (indicating “very much”). The Likert scales were used as an answer rating for the self-constructed questions, as it generally has good validity.^32^

After receiving the IN interventions, participants were surveyed 24 hours later to assess the improvement of their reported symptoms within that period. They were asked to rate the extent of improvement on a 5-point Likert scale ranging from 1 (indicating “not at all”) to 5 (indicating “very much”), based on their baseline survey. And they rated their sleep quality over the last previous 24 hours, on a 4-point Likert scale from “very bad” to “very good.” Furthermore, satisfaction, perceptions and experiences toward IN were assessed through a set of 9 items. The patient questionnaire included questions to evaluate the IN, the impact on well-being, the impact on complaints, the additional physical and psychological burden caused by IN, the inpatient stay, the benefit of IN, and the desire to continue of the IN offer.

Additionally, a comprehensive documentation of consultations and IN interventions was established, capturing information such as the type of IN interventions, body region, oil used, and duration of the intervention.

### Statistical Analyses

IBM SPSS Statistics, version 28.0 was utilized for all data analyses in this small-scale pilot study. Given the exploratory nature of the study and the limited sample size, the analyses remained descriptive, focusing on absolute and relative frequencies, without inferential testing. The standardized survey instruments were analyzed according to the recommended guidelines from the validation publications.^24,30,31^

## Results

During the 6-month study period, the IN consultation was requested by medical and nursing staff for 66 patients, with 4 patients declining the offer of IN. This resulted in an acceptance rate of 93.9%. Out of the 62 patients who received IN, 21 patients (33.9%) participated in the study. The primary reason for non-participation was the presence of non-inclusion criteria, such as severe cognitive deficits or limited communication skills. Among the 21 study participants, 13 (61.9%) were able to complete the questionnaire independently, while the remaining participants required assistance from the study staff. Follow-up questionnaires were returned by 20 out of 21 patients (95.2%). A total of 36 IN interventions were conducted for the 21 study participants during the 24-hour survey period. Notably, no relevant allergies were named by the patients and no adverse events were reported throughout the study period.

### Characteristics of Study Participants

The sociodemographic characteristics of the participants are presented in Table 2. The mean age of the participants was 50.8 ± 15.9 years, ranging from 24 to 82 years. Among them, 15 (71.4%) were female, and 9 (42.9%) were either married or cohabiting. Notably, all participants had an oncologic diagnosis, with approximately three-quarters of them being diagnosed with lymphoma.

**Table 2.** Characteristics of the Study Participants (n = 21).

Characteristic		Mean ± SD n (%)
Age		50.8 ± 15.9
Sex	Female	15 (71.4)
	Male	6 (28.6)
Marital status	Single	7 (33.3)
	Married, cohabiting	9 (42.9)
	Widowed	3 (14.3)
	Divorced	2 (9.5)
Vocational degree	No	3 (14.3)
	Yes	18 (85.7)
Ward	Oncology ward	19 (90.5)
	Palliative ward	2 (9.5)
Cancer type	Lymphoma	15 (71.4)
	Other	6 (28.6)

### Integrative Nursing Interventions

Among the 21 study participants, a total of 189 IN interventions were conducted during their entire hospital stay. However, 36 IN interventions were carried out as part of the 24-hour survey period: The number of IN interventions received per participant ranged from 1 to 3. The average duration of an IN intervention was 23 ± 11.0 minutes, with a minimum of 15 minutes and a maximum of 45 minutes. Length of time was dependent on type of IN intervention. Table 3 provides an overview of the body region treated, oils used, and types of IN intervention. The most treated body region was the feet/legs (n = 18; 50.0%), followed by the back (n = 9; 25.0%). Solum oil (n = 15; 41.7%) and lavender oil (n = 5; 13.9%) were the most frequently used oils. Embrocation (n = 28 77.8%) was the predominant type of IN intervention.

**Table 3.** Body Region, Used Oils and Type of the IN Interventions (n = 36).

		n (%)
Body region	Feet/legs	18 (50.0)
	Back	9 (25.0)
	Abdomen	5 (13.9)
	Arms/hands	3 (8.3)
	Chest	1 (2.8)
Used oil	Solum	15 (41.7)
	Lavender	5 (13.9)
	Yarrow (tea infus)	5 (13.9)
	Rosemary	3 (8.3)
	Aconite	2 (5.6)
	Citrus	2 (5.6)
	Thyme	2 (5.6)
	Mallow	1 (2.8)
	Rose	1 (2.8)
Type of IN intervention	Embrocation	28 (77.8)
	Compress	5 (13.9)
	Foot/hand bath	3 (8.3)

### Survey Data

The results of the baseline survey (t0) are presented in Table 4. The mean FACIT-F score was 29.2 ± 12.5 (min: 5, max: 46). Regarding the PHQ-4 score, the mean was 3.0 ± 1.9 (min: 0, max: 6). Among the participants, 8 (38.1%) had normal scores, 11 (52.4%) had mild elevations, 2 (9.5%) had moderate elevations, and none had severe elevations. 4 participants (19.0%) exhibited probable anxiety, while 6 (28.6%) exhibited probable depression. Overall, 9 participants (42.9%) rated their subjective health positively.

**Table 4.** Baseline Survey Data (t0).

		Mean ± SD n (%)	Median (range)
FACIT-F (n = 18)	Fatigue	29.2 ± 12.5	28 (5-46)
PHQ-4 (n = 21)	Psychological burden	3.0 ± 1.9	3 (0-6)
GAD-2 (n = 21)	Absence of probable anxiety	17 (81.0)	
	Presence of probable anxiety	4 (19.0)	
PHQ-2 (n = 21)	Absence of probable depression	15 (71.4)	
	Presence of probable depression	6 (28.6)	
Mobility (n = 20)		5.3 ± 3.3	5.5 (0-9)
Life satisfaction (n = 20)		7.1 ± 2.7	8 (0-10)
Subjective health (n = 21)	Very good	0 (0.0)	
	Good	9 (42.9)	
	Fair	7 (33.3)	
	Bad	5 (23.8)	
	Very bad	0 (0.0)	
Sleep quality over the last 4 wks (n = 21)	Very good	1 (4.8)	
	Rather good	7 (33.3)	
	Fairly bad	12 (57.1)	
	Very bad	1 (4.8)	
Pain (n = 21)	No	13 (61.9)	
	Yes	8 (38.1)	
Further symptoms (n = 19-21)	Sleep disorders	3.0 ± 1.4	3 (1-5)
	Other side effects of cancer therapy	2.7 ± 1.2	3 (1-5)
	Restlessness	2.4 ± 1.1	2 (1-5)
	Edema	2.1 ± 1.2	2 (1-5)
	Digestive problems	2.1 ± 1.5	1 (1-5)
	Respiratory complaints	1.8 ± 1.3	1 (1-5)
	Panic attacks, rapid heartbeat	1.8 ± 1.2	1 (1-5)

In terms of sleep quality over the past 4 weeks, 13 participants (61.9%) described it as rather or very poor. The primary reasons reported for this poor subjective sleep quality were waking up at night or early in the morning (n = 10) and getting up to go to the toilet (n = 6; multiple answers possible). Additionally, 8 patients (38.1%) reported experiencing physical pain. Furthermore, participants ranked sleep disorders (3.0 ± 1.4), side effects of tumor therapy (2.7 ± 1.2), and restlessness (2.4 ± 1.1) as the most common and severe symptoms.

Table 5 presents the results of the surveys conducted 24 hours after the IN intervention (t1). Regarding sleep quality, 17 patients (85.0%) reported it as rather or very good for the previous night. Participants were asked to rate the improvement of their symptoms over the past 24 hours compared to the baseline survey. The greatest improvements were reported for restlessness (3.4 ± 1.2), respiratory complaints (3.1 ± 1.8), and sleep disorders (2.9 ± 1.5).

**Table 5.** Data From the Survey 24 Hours After the IN Intervention (t1).

		Mean ± SD n (%)	Median (range)
Sleep quality over the past 24 h (n = 20)	Very good	4 (20.0)	
	Rather good	13 (65.0)	
	Fairly bad	2 (10.0)	
	Very bad	1 (5.0)	
Symptom relief in the past 24 h (n = 7-14^a^)	Restlessness	3.4 ± 1.2	4 (1-5)
	Respiratory complaints	3.1 ± 1.8	4 (1-5)
	Sleep disorders	2.9 ± 1.5	3 (1-5)
	Digestive problems	2.9 ± 1.3	3 (1-4)
	Other side effects of cancer therapy	2.7 ± 1.2	2.5 (1-5)
	Edema	2.6 ± 1.2	2.5 (1-5)
	Panic attacks, rapid heartbeat	2.4 ± 1.1	2 (1-4)

a After receiving the IN intervention, participants were surveyed 24 hours later to assess the improvement of their reported symptoms within that period. They were asked to rate the extent of improvement on a 5-point Likert scale ranging from 1 (indicating “not at all”) to 5 (indicating “very much”), based on their baseline survey.

In terms of additional burden due to the IN interventions, the majority of patients (n = 17; 85.0%) did not report any emotional or physical burden. The evaluation of IN interventions was predominantly positive, with 4 (20.0%) rating it as rather positive and 16 (80.0%) as very positive. Regarding the effect on well-being, 15 (75.0%) reported a very positive effect, 4 (20.0%) reported a somewhat positive effect, and 1 (5.0%) reported no effect. Additionally, 16 respondents (80.0%) perceived relief of their symptoms, while 3 (15.0%) reported no relief. Almost all patients (n = 19; 95.0%) expressed a desire to receive IN as part of their future hospitalization.

## Discussion

The present study has demonstrated the use of IN interventions on cancer-related symptoms in inpatients on 2 oncology wards. IN provides an opportunity to support patients with non-pharmacological interventions during their inpatient stay.

In general, patient acceptance of IN was high. Almost all patients—62 of the 66 patients (93.9%) referred to the IN consultation by nursing and medical staff—accepted the supportive care offered by IN. The high level of acceptance demonstrated the openness and interest in IN within the project. However, there was a notable discrepancy between the high patient acceptance of IN with 93.9% and the low participation rate in the present study with 33.9%, which needs to be discussed.

The 2 project wards were, on the 1 hand, a regular oncology ward with patients mostly undergoing curative treatment and, on the other hand, a palliative care ward. Consequently, the patient population consisted mainly of seriously ill cancer patients. The results and the acceptance rate suggest a great need for IN among these patients. At the same time, this may be a reason for the low participation rate in the present study. Due to the fact that the patients were severely debilitated by cancer and/or cancer treatment, it was difficult for them to participate in written surveys during their hospitalization. Patients could not be included in the study because they met the non-inclusion criteria or because did not feel able to complete multi-page questionnaires at 2 points in time. Even among those who participated, almost half of the patients needed assistance to complete the questionnaire. The present study was also an accompanying research, so patients could participate in the project and the IN consultation even without participating in the study. In future studies, measures should be implemented to facilitate easy and accessible participation in surveys, ensuring a broader representation of patient perspectives.

The findings of the present study support the well-established notion that oncology patients experience significant physical and mental burden,^33^ including during their inpatient stay.^33-35^ Existing studies have consistently shown that these patients suffer from fatigue, sleep disorders, psychological stress, and other distressing symptoms.^36,37^ For instance, the average FACIT-F score in the general population is reported to be 43.5,^24^ which is notably higher than the score of 29.2 observed in the present survey, indicating comparatively poor patient performance. This aligns with the results of a previous study on lung cancer patients, where the FACIT-F score was found to be 30.0.^38^

Moreover, the average PHQ-4 score in this study was 3.0, higher than the score of 2.5 reported in the general population.^30^ Other studies on cancer patients have also highlighted an increased psychological burden and the need for support.^29,35^ In comparison to the normal German population, the patients in the present study were twice as likely to exhibit signs of anxiety disorder and 3 times as likely to display signs of depressive disorder.^39^

Furthermore, a review by Divani et al underscores the deterioration of sleep quality during cancer treatment, emphasizing the necessity for specific and preventive interventions.^40^ Consistent with these findings, sleep disorders were reported as the most prevalent and severe symptom among the patients in the present study. Accordingly, sleep problems appear to be a significant issue for oncology patients and should be emphasized.^41^

In a study on nurse-guided patient self-treatment in integrative oncology, Ben-Arye et al demonstrated symptom improvement within 24 hours of intervention as a short-term effect.^42^ Many participants in the present study have experienced notable improvements in their overall well-being and specific symptoms. The offer of IN was positively evaluated by the participants: The satisfaction levels with the IN were remarkably high, with a large proportion of participants reporting a positive experience. These findings provide initial indications suggesting that IN may offer valuable benefits to oncology inpatients. To mitigate potential social desirability bias, future studies should include both within-group and between-group comparisons to provide more comprehensive assessment of the intervention’s effectiveness.

## Limitations

Descriptive pilot studies naturally have several limitations that should be considered when interpreting the findings. First, the small sample size of 21 patients restricts the generalizability of the results. Second, the study was conducted at a single university hospital, which may limit the transferability of the findings. Third, as participation in this study is voluntary and a convenience sampling is used, there may be a potential recruitment bias and simultaneous selection bias. The data collected relied on self-report measures, introducing the possibility of response bias, recall bias, and social desirability bias. Furthermore, the study utilized a pre-post design without a control group, making it difficult to put the data in context. Moreover, the short-term follow-up period of approximately 24 hours does not capture the longer-term effects of IN on patient outcomes. Last, the study lacked qualitative data, which could have provided deeper insights into patient experiences. These limitations highlight the need for further research with larger and more diverse populations, rigorous study designs, and longer-term follow-up to strengthen the evidence base and fully understand the implementation and effectiveness of IN in improving patient outcomes.

IN shows great potential in providing valuable support to hospitalized patients. While IN has traditionally been practiced based on internal evidence such as experiential knowledge and expertise, it is crucial to subject it to scientific investigation.^43^ Building on the findings of this descriptive pilot study, the IMPLEMENT-UKU project aims to implement a multimodal approach to IN across additional wards and evaluate its implementation, execution and effectiveness using a robust research design. The goal is to generate meaningful insights that can inform the future utilization of IN interventions and further enhance patient care.

## Conclusion

In conclusion, the findings of the present study suggest that there is an openness and interest in IN interventions among inpatients on oncology wards. IN is evaluated positively by patients with good perceptions and experiences. The study highlights the potential of IN as a supportive care intervention, offering additional benefits to patients during their inpatient stay. Moving forward, further comprehensive studies are required to assess the specific effects of IN interventions and to investigate its effectiveness in a multimodal or complex framework and in a comparative setting.
